# Feasibility of fully automated closed-loop glucose control using continuous subcutaneous glucose measurements in critical illness: a randomized controlled trial

**DOI:** 10.1186/cc12838

**Published:** 2013-07-24

**Authors:** Lalantha Leelarathna, Shane W English, Hood Thabit, Karen Caldwell, Janet M Allen, Kavita Kumareswaran, Malgorzata E Wilinska, Marianna Nodale, Jasdip Mangat, Mark L Evans, Rowan Burnstein, Roman Hovorka

**Affiliations:** 1 Wellcome Trust-MRC Institute of Metabolic Science, Metabolic Research Laboratories, University of Cambridge, Addenbrooke's Hospital, Hills Road, Cambridge, CB2 0QQ, UK; 2 Neurosciences Critical Care Unit, Addenbrooke's Hospital, Hills Road, Cambridge, CB2 0QQ, UK

## Abstract

**Introduction:**

Closed-loop (CL) systems modulate insulin delivery according to glucose levels without nurse input. In a prospective randomized controlled trial, we evaluated the feasibility of an automated closed-loop approach based on subcutaneous glucose measurements in comparison with a local sliding-scale insulin-therapy protocol.

**Methods:**

Twenty-four critically ill adults (predominantly trauma and neuroscience patients) with hyperglycemia (glucose, ≥10 m*M*) or already receiving insulin therapy, were randomized to receive either fully automated closed-loop therapy (model predictive control algorithm directing insulin and 20% dextrose infusion based on FreeStyle Navigator continuous subcutaneous glucose values, *n *= 12) or a local protocol (*n *= 12) with intravenous sliding-scale insulin, over a 48-hour period. The primary end point was percentage of time when arterial blood glucose was between 6.0 and 8.0 m*M*.

**Results:**

The time when glucose was in the target range was significantly increased during closed-loop therapy (54.3% (44.1 to 72.8) versus 18.5% (0.1 to 39.9), *P *= 0.001; median (interquartile range)), and so was time in wider targets, 5.6 to 10.0 m*M *and 4.0 to 10.0 m*M *(*P *≤ 0.002), reflecting a reduced glucose exposure >8 and >10 m*M *(*P *≤ 0.002). Mean glucose was significantly lower during CL (7.8 (7.4 to 8.2) versus 9.1 (8.3 to 13.0] m*M; P *= 0.001) without hypoglycemia (<4 m*M*) during either therapy.

**Conclusions:**

Fully automated closed-loop control based on subcutaneous glucose measurements is feasible and may provide efficacious and hypoglycemia-free glucose control in critically ill adults.

**Trial Registration:**

ClinicalTrials.gov Identifier, NCT01440842.

## Introduction

Abnormalities of glucose metabolism are common in critically ill patients [1,2] and are characterized by hyperglycemia [3-5], hypoglycemia [6,7], and increased glucose variability [8,9], each independently and additively associated with higher adjusted mortality rates [10]. Mechanisms of this adversity are not fully understood but may be related to increased susceptibility to sepsis, endothelial dysfunction, increased oxidative stress, and predisposition to cardiac arrhythmias [6,11].

The extent to which hyperglycemia in critical illness should be corrected has been the focus of number of prospective studies [12-16] with conflicting results and remains the subject of an ongoing debate [17]. Possible explanations are different glucose targets in the control groups, different types of devices for blood-glucose measurement, as well as different nutritional strategies and varying levels of expertise with insulin therapy among the intensive care nurses [18].

Hypoglycemia is associated with adverse outcomes and may have negated any beneficial effect from intensive glucose control in those patients in whom target glucose levels were achieved.

Existing tools for achieving desired glucose levels range from sliding and dynamic scales, and paper-based protocols to computerized protocols that advise the nursing staff [19]. Safe implementation of insulin therapy requires accurate and frequent glucose measurements, but even hourly glucose measurements may fail to identify hypoglycemia during periods of rapid glucose change. Further, frequent sampling may be inconvenient for the patient and adds to the workload of the nursing staff [20].

Over the last decade, continuous subcutaneous glucose monitoring (CGM) has emerged as a valuable tool in the management of diabetes [21,22]. A number of studies have investigated the accuracy of CGM devices in critical illness and have reported acceptable CGM performance [23-25], but the clinical efficacy and effectiveness of CGM devices in daily-life ICU practice is not yet established.

Availability of reliable continuous subcutaneous glucose monitoring has led to a rapid expansion of research into closed-loop insulin delivery, documenting superior performance compared with conventional pump therapy in type 1 diabetes [26].

The objective of the present study was to investigate the feasibility of automated closed-loop glucose control based on continuous subcutaneous glucose measurements in critically ill adults.

## Materials and methods

### Patients and study design

The study was an investigator-initiated, prospective single-center randomized controlled parallel-group open-label trial performed at the 24-bed Neurosciences Critical Care Unit (NCCU) at Addenbrooke's Hospital, Cambridge, UK, a tertiary trauma and neurosurgical referral center in the East of England with approximately 900 admissions per year (90% trauma or neurosciences patients). A separate research nurse was responsible for all study-related activities. Cambridge Central Research Ethics Committee approved the study.

Study participants were recruited from May 2012 to September 2012. All critically ill patients consecutively admitted to NCCU were screened for eligibility. Inclusion criteria were age 18 years and older, stay at NCCU expected of at least 48 hours, and arterial glucose level greater than 10.0 m*M *or already receiving insulin treatment, including preexisting diabetes. Exclusion criteria were diabetic ketoacidosis or hyperosmolar state, therapeutic hypothermia, known or suspected allergy to insulin, fatal organ failures, significant abnormalities of blood clotting, pregnancy, and treatment with external cardiac pacemaker.

Written informed consent/assent was obtained before enrolling a patient in the study, either from the patient, or, if patients lacked capacity, from the next of kin. Patients entered into the trial were randomized to an automated closed-loop or local sliding-scale insulin-therapy protocol by using the minimization method [27], implemented in the Minim program [28] to balance between group characteristics: Acute Physiology and Chronic Health Evaluation II (APACHE II) score, glucose at the time of randomization, body mass index, and preexisting diabetes. Randomization was carried out at the time of recruitment by the investigator by using a dedicated study laptop.

### Common study procedures

Apart from glucose control, all other aspects of patient care, including nutritional management and treatment of hypoglycemia and hyperglycemia, were carried out according to local treatment protocols and were identical between treatment arms. Actrapid insulin (Novo Nordisk, Bagsværd, Denmark), in a concentration of 50 U in 50 ml of 0.9% saline, was used in both treatment arms. All study-related activities were carried out for a maximum period of 48 hours or until the end of the NCCU stay, whichever came first.

The study was terminated if the subject was moved out of NCCU for more than 2 hours.

### Automated closed-loop therapy

**Figure 1:**
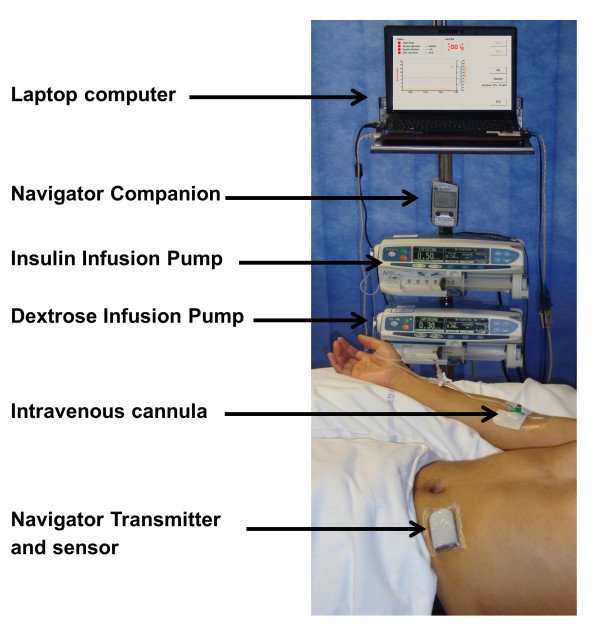
**Components of the closed-loop glucose-control system**.

**Figure 2:**
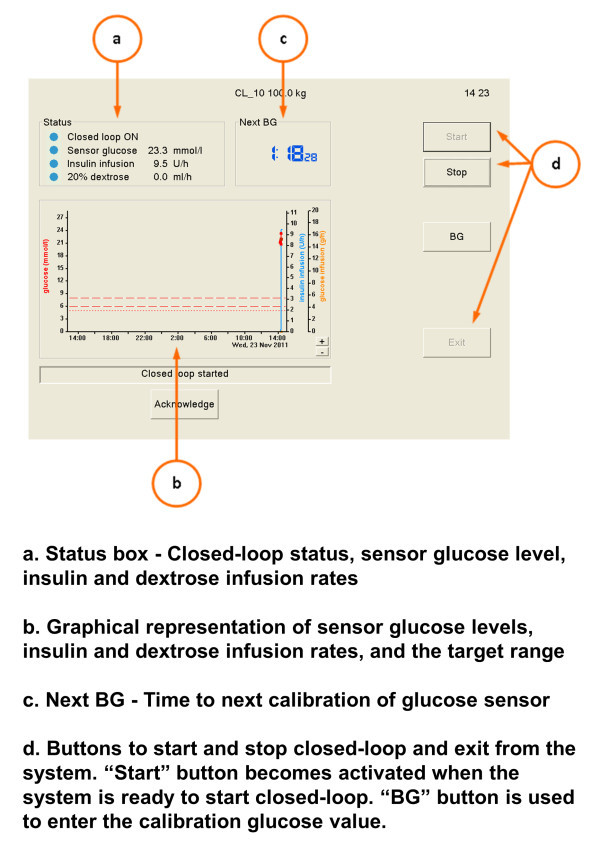
**User interface of the closed-loop system**.

Subjects randomized to closed-loop therapy were treated by using an automated closed-loop system comprising (a) FreeStyle Navigator subcutaneous continuous glucose-monitoring system (Abbott Diabetes Care, Alameda, CA, USA), (b) a laptop computer running a model predictive control (MPC) algorithm, and (c) two Alaris CC Plus syringe pumps (CareFusion, Basingstoke, UK) (Figure 1). The CGM system uses CE-marked FreeStyle Navigator Transmitter, and a non-CE-marked investigational receiver device Navigator Companion (Abbott Diabetes Care), equivalent in its function and calibration algorithm to CE-marked Navigator Receiver with a 1-hour warm-up time [29]. The sensor was inserted in either the anterior abdominal wall or the upper arm. The user interface is shown in Figure 2.

We used a control algorithm based on the model predictive control approach [30], optimized and tuned *in silico *by using a computer-simulation environment validated for glucose control in the critically ill [31]. Every 5 minutes, the algorithm calculated insulin or, at low glucose values, 20% dextrose-infusion requirements based on minute-by-minute real-time sensor glucose values. The insulin and dextrose pumps were controlled automatically, and no manual intervention was required. The calculations used a compartment model of glucose kinetics [32], describing the effect of insulin on sensor glucose excursions. The algorithm was initialized by using patient's weight and adapted itself to a particular patient by updating two model parameters: a rapidly changing glucose flux correcting for errors in model-based predictions, and a slowly changing estimate of an insulin rate to maintain euglycemia. The individualized model forecasted plasma glucose excursions over a 1- to 1.5-hour prediction horizon when calculating the insulin rate and a 30- to 40-minute horizon when calculating the dextrose rate. Safety rules limited maximum insulin (50 U/h) and 20% dextrose (200 ml/h) delivery and prevented insulin delivery at sensor glucose below 1.2 m*M *of the target glucose level. Information about enteral or parenteral nutrition was not provided to the algorithm. The algorithm requested a reference glucose measurement every 1 to 6 hours (at a sensor level below 3.5 m*M *every 30 minutes); frequency depended on the deviation between sensor and reference glucose values. Reference glucose was used to recalibrate the sensor and to direct insulin and dextrose delivery when sensor levels were not available, such as during the 1-hour warm-up period. We used icuMPC algorithm version 1.0.6.

### Local insulin therapy protocol

Subjects allocated to the local insulin therapy protocol followed the usual care of a paper-based intravenous insulin-administration protocol used in NCCU (Table 1). When the patient's glucose control was deemed unsatisfactory, the bedside nurse could initiate a physician-prescribed alteration in the paper-based scale either to increase or to decrease the amount of insulin delivered for a given glucose level, as per usual practice. Similarly, insulin or dextrose boluses were prescribed at the discretion of the treating physician.

**Table 1:** Local intravenous insulin titration protocol

Blood glucose (m*M*)	Insulin infusion rates^a ^(Units/hour)
20.0	6.0, inform physician**^b^**
17.1-20.0	4.0, inform physician**^b^**
14.1-17.0	3.0, inform physician**^b^**
11.1-14.0	2.5, inform physician**^b^**
8.6-11.0	2.0, inform physician**^b ^**if glucose >10
7.1-8.5	1.5
5.6-7.0	1.0
4.0-5.5	0.5
<4.0	NIL, inform physician**^b^**

a50 Units Insulin Actrapid in 50 ml of 0.9% saline. bTarget glucose was 7 to 10 m*M*, and when the glucose was outside this target, hourly infusion rates were adjusted by the attending physician.

### Reference glucose measurements

Arterial blood glucose measurements were made by using an on-site blood gas analyzer (Cobas b 221; Roche Diagnostics, Burgess Hill, UK) at hourly intervals. As previously described in the investigational arm, a subset of reference glucose values was provided as the algorithm dictated, but the remainder of the reference samples did not factor into patient management.

In the control arm, however, the hourly reference glucose values were available to the clinical team for insulin-dose adjustments.

### Assessments and data collection

Demographic and clinical characteristics, including APACHE II scores, were collected at study initiation. Patients were classified as having diabetes on the basis of medical history. Treatment with corticosteroids and inotropes was defined as treatment with these agents during any part of the study, including those subjects already taking these agents at study entry. From the time of randomization to the time of discharge from the ICU or 48 hours after randomization, whichever came first, we recorded all blood glucose measurements, insulin administration, type and volume of all enteral and parenteral nutrition and additional intravenous glucose administered, and corticosteroid and inotrope administration.

### Statistical analysis

Investigators agreed on the outcome measures and the analysis plan in advance. The primary outcome was the time spent in primary target-glucose range between 6.0 and 8.0 m*M*, as recorded by reference glucose measurements. Secondary efficacy outcomes were time spent with glucose levels between 4.0 and 10.0 m*M*, between 5.6 and 10.0 m*M*, above and below target ranges, mean and standard deviation of reference glucose, sensor accuracy metrics, and insulin-infusion rates. Safety end points included frequency and magnitude of significant hypoglycemic (<3.0 m*M *and <2.0 m*M*) and significant hyperglycemic (>15 and 17 mM) episodes and other adverse events. Utility end points included the number of the reference glucose values requested by the algorithm and CGM availability.

As this was a feasibility study, no formal power calculations were performed. All analyses were performed on an intention-to-treat basis. An unpaired *t *test was used to compare normally distributed variables. Nonnormally distributed variables were compared by using a Mann-Whitney *U *test. Calculations were carried out by using SPSS Version 19 (IBM Software, Hampshire, UK). Outcomes were calculated with GStat software, Version 1.3 (University of Cambridge, UK). Values are given as mean (SD) or median (interquartile range). A *P *value <0.05 was considered statistically significant.

## Results

### Study participants

In total, 37 patients were screened. The next-of-kin refused consent in seven patients, and three patients failed inclusion/exclusion criteria. Of the 27 randomized subjects, two subjects left the intensive care unit within 24 hours of the study start, and one subject was initiated on therapeutic hypothermia within 24 hours. Efficacy but not safety data from these three subjects were excluded from the data analysis.

Twenty-four recruited subjects were analyzed (12 closed-loop and 12 local protocol); 21 (88%) subjects completed the intended 48 hours, whereas the remaining three (12%) subjects completed 24, 34, and 41 study hours because of early discharge from the NCCU. The baseline characteristics of the two groups were similar (Table 2), with comparable APACHE Il scores, previous diabetes status, and body mass index. Of the 24 subjects, 11 (46%) had a history of preexisting diabetes. The majority (83%) of participants were already receiving insulin infusion at the time of study enrolment. The proportion of postsurgical patients was similar between two groups, whereas patients with major trauma were more common in the closed-loop group.

**Table 2:** Baseline characteristics, nutritional intake, and corticosteroid and inotrope treatment of the study population

	Local protocol (*n *= 12)	Automated closed-loop (*n *= 12)
Age (years)	58.3 ± 12.5	62.8 ± 16.0
Male sex (*n*/%)	9 (75%)	9 (75%)
White ethnicity	11 (92%)	12 (100%)
Weight (kg)	83.5 (80.0-89.2)	81.4 (62.5-97.5)
BMI	27.8 (25.9-30.8)	27.1 (26.4-31.4)
APACHE II score at randomization	11.2 ± 3.4	12.9 ± 5.0
Highest APACHE II score first 24 hours of admission	13.8 ± 5.0	16.2 ± 5.4
Time between admission and study start (days)	2 (1-7)	1 (1-3)
Previous diabetes	6 (50%)	5 (42%)
Insulin infusion at study start	10 (83%)	10 (83%)
Reason for ICU admission		
Medical	1 (8%)	3 (25%)
After neurosurgery	4 (33%)	4 (33%)
Trauma	7 (58%)	5 (42%)
Total energy (kcal/hour)	66.4 (17.2)	60.0 (18.4)
Total CHO (g/hour)	7.9 (1.6)	7.1 (2.2)
Feeding interruptions/day	1 (0-1.5)	2 (0-2)
Corticosteroid treatment	3 (25%)	5 (42%)
Inotrope treatment	4 (33%)	5 (42%)

Data shown are mean ± SD, median (interquartile range), or number (%). APACHE II, Acute Physiology and Chronic Health Evaluation II; BMI, body mass index.

### Glucose control and insulin and dextrose administration

**Figure 3:**
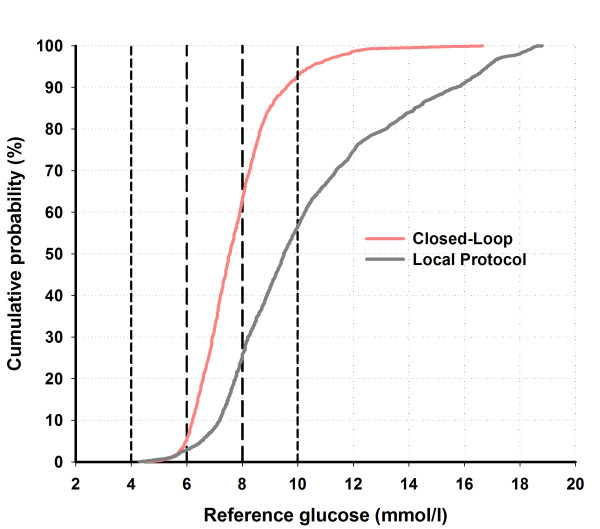
**Cumulative distribution of reference glucose values obtained during closed-loop and local treatment protocol**. Dashed vertical lines indicate the primary study target range from 6.0 to 8.0 m*M*. Vertical fine dashed lines indicate the wider target from 4.0 to 10.0 m*M*.

**Figure 4:**
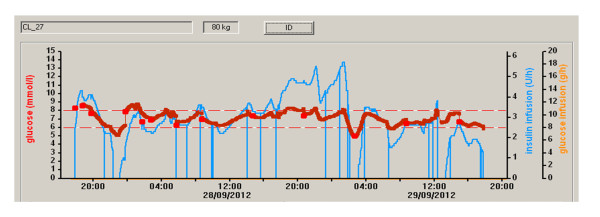
**An example of the 48-hour closed-loop study**. Darker red continuous line represents sensor glucose. Lighter red squares represent reference glucose measurements used for sensor calibration. Blue line represents insulin infusion. Thin red dashed lines indicate primary target. Dextrose infusion was not required in this study.

The time spent in the primary target glucose range (6.0 to 8.0 m*M*) was significantly higher during closed-loop therapy (54.3% (44.1 to 72.8) versus 18.5% (0.1 to 39.9), closed-loop versus local protocol, *P *= 0.001, median (interquartile range), Table 3). These differences were more pronounced during the first 24 hours, with a fourfold improvement of time spent in the target glucose range (59.4% (49.0 to 71.1) versus 14.5% (0.0 to 34.5), *P *= 0.001). These results persisted when the time was spent in a wider target range of 4.0 to 10.0 m*M *and 5.6 to 10.0 m*M *(Table 3). Time spent at greater than 8.0 m*M *and 10.0 m*M *was significantly lower during closed-loop therapy. The cumulative distributions of glucose values during closed-loop therapy and the local protocol are shown in Figure 3, documenting comparable frequency of glucose levels <5 m*M*. A sample 48-hour closed-loop study is shown in Figure 4.

**Table 3:** Results based on reference glucose and insulin-infusion data

	Local protocol (*n *= 12)	Automated closed-loop (*n *= 12)	*P*
Primary end point			
Time glucose in target (%) (6.0-8.0 m*M*)	18.5 (0.1-39.9)	54.3 (44.1-72.8)	0.001
			
Secondary end points			
Starting glucose (m*M*)	10.8 (9.9-12.0)	10.0 (8.9-11.1)	0.21
Mean glucose (m*M*)	9.1 (8.3-13.0)	7.9 (7.4-8.2)	0.001
Standard deviation of glucose (m*M*)	1.9 (0.8)	1.3 (0.5)	0.089
Time spent at glucose levels (%)			
4.0-10.0 m*M*	73.2 (21.2-89.4)	93.3 (86.5-100.0)	0.002
5.6-10.0 m*M*	73.2 (21.2-82.4)	92.2 (83.4-99.2)	0.001
>8.0 m*M*	78.4 (57.6-99.9)	39.0 (23.5-51.4)	0.001
>10.0 m*M*	26.8 (10.5-78.8)	6.7 (0-13.5)	0.002
<6.0 m*M*	0 (0-3.0)	4.6 (3.1-8.3)	0.028
<5.6 m*M*	0 (0-0)	0.7 (0-2.7)	0.128
<4.0 m*M*	0 (0-0)	0 (0-0)	NA
Hypoglycemia			
Episodes <4.0 m*M *	None	None	
Hypoglycemia treatments	None	None	
Hyperglycemia			
Number of subjects ≥15 m*M*	5 (42%)	1 (8%)	
Number of subjects ≥17 m*M*	4 (33%)	1 (8%)	
Episodes ≥15 m*M *	11	1	
Episodes ≥17 m*M*	13	1	
Insulin-infusion data			
Total units for 24 hours	40.9 (34.9-101.4)	57.4 (40.0-112.2)	0.478
Hourly infusion rate	1.7 (1.5-4.2)	2.4 (1.7-4.7)	0.478
Total dextrose infusion for 48 hours (g)	0.21 (0.0-5.2)	NA	NA

Data shown are mean (SD) or median (interquartile range).

**Figure 5:**
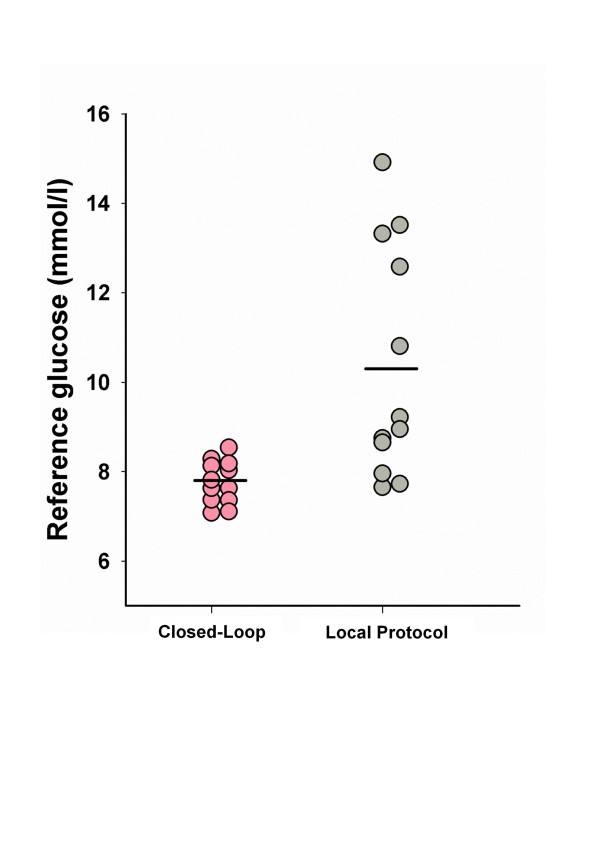
**Mean reference glucose per subject during closed-loop (*n *= 12) and local treatment protocol (*n *= 12)**. Horizontal black line indicates the mean reference glucose in each intervention arm.

**Figure 6:**
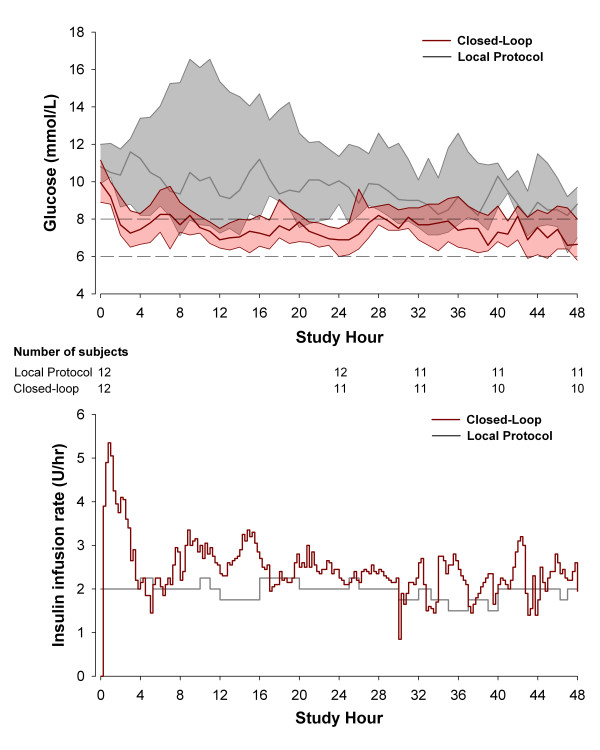
**Glucose and insulin values during infusion**. **Top panel: **Glucose profiles (median and interquartile range) during closed-loop and local treatment protocol. **Bottom panel: **Median insulin infusion rates during closed-loop and local treatment protocol. The dashed lines indicate the primary target range from 6 to 8 m*M*.

The mean glucose level was significantly lower during closed-loop therapy (7.9 (7.4 to 8.2) versus 9.1 (8.3 to 13.0) m*M; P *= 0.001) and more consistent among subjects in comparison to the local protocol (Figure 5). Glucose variability assessed by the standard deviation tended to be lower during the closed-loop therapy, without reaching statistical significance. Reference glucose profiles shown in Figure 6 highlight differences between the two groups. The closed-loop system administered more insulin during the first study hours (Figure 6, bottom panel), but overall, no statistical difference was found in insulin infusion between the treatments (Table 3). During closed-loop therapy, six (50%) of 12 patients received 20% dextrose, with a total amount less than 10 g per 24 hours, and one patient (8%) received 28 g dextrose per 24 hours.

### Nutrition and concomitant treatment

All but one patient received enteral nutrition, according to the local NCCU protocol. One patient received both enteral and parenteral nutrition. The number of calories and carbohydrates as well as the number of feeding interruptions per day was comparable between the two interventions (Table 2). The proportion of patients treated with steroids or inotropes during the 48-hour study period was slightly higher during closed-loop therapy (Table 2).

### Safety

No hypoglycaemic events (<4.0 m*M*) or other adverse events occurred in either group. The numbers of patients and the numbers of episodes with glucose >15 and 17 m*M *were higher during treatment with the local protocol.

### Utility assessment and sensor performance

During closed-loop therapy, the number of reference glucose measurements requested by the control algorithm was 9.5 (9.0 to 14.0) during the first 24 hours and 7.0 (4.0 to 8.0) during the second 24 hours. This translated into an interval between sensor calibrations of 152 (105 to 160) and 205 (180 to 360) minutes during the first and second 24 hours, respectively. Sensor performance was good, with the median absolute deviation of 0.5 (0.3 to 1.0) m*M*, median relative absolute deviation of 7.0% (3.5 to 13.0), with 87.8% of sensor values within 20% of reference glucose. When the sensor levels were not available, the control algorithm directed insulin/dextrose delivery based on hourly reference glucose measurements, which were manually put into the algorithm. Overall, sensor unavailability for the entire 48-hour study period during closed-loop therapy was 25 (0 to 207) minutes. This translated to 5.6% of the closed-loop period, including the first hour of the study, during which the sensor was warming up. Excluding the mandatory first-hour sensor warm-up period, 3.4% of the closed-loop period used reference glucose values manually input. This occurred mostly during the first 10 hours of sensor use. Two subjects required replacement of sensor because of MRI scanning.

## Discussion

We documented that automated closed-loop glucose control, based on continuous subcutaneous glucose levels, is feasible and may significantly improve glucose levels without increasing the risk of hypoglycemia in critically ill adults. Compared with local intravenous sliding-scale therapy, closed-loop therapy increased up to fourfold the time spent in the target glucose range and reduced the time spent at higher glucose levels. Subjects treated with closed-loop therapy achieved consistent results, with a trend toward reduced glucose variability without requiring nurse interventions or decision making on insulin delivery.

Reflecting the current practice recommendations for glucose control in the intensive care unit [33,34], we adopted a moderate glucose target of 6.0 to 8.0 m*M *rather than the tight glycemic range 4.4 to 6.1 m*M *of the Leuven and NICE-SUGAR studies. The upper limit of our target range is similar to recent consensus guidelines (<8.3 m*M*) [35]. Based on our simulation work, we were confident of achieving a target between 6.0 and 8.0 m*M *without increasing the risk of hypoglycemia.

Subjects in the local-treatment protocol were treated with an intravenous sliding-scale protocol intended to maintain glucose in a safe target range of 7 to 10 m*M *without increasing the risk of hypoglycemia. We did not change the target range of the usual treatment for two reasons. First, we aimed to compare current local practice with a novel treatment; second, we could not guarantee patient safety by changing the target range of the sliding-scale protocol. The mean glucose level achieved during closed-loop control was 7.8 m*M *and was within the range associated with the lowest mortality in observational studies [5,36]. Importantly, during the present study, closed-loop therapy achieved safe glucose levels without increasing the risk of hypoglycemia. Glucose variability, as measured by the standard deviation, tended to be lower during closed-loop without reaching statistical significance. Because both hypoglycemia and glucose variability have been associated with adverse outcomes, beneficial effects, apart from glucose lowering, may be achieved with closed-loop therapy.

Since the introduction of intensive insulin therapy, different algorithms and control systems aiming at effective and safe glucose control have been proposed [19]. These can range from written guidelines [12,13] and protocols [37-40] to elementary [41,42] and advanced computerized algorithms [43-48]. We used an advanced computer algorithm belonging to the family of model predictive control. The control algorithm and calibration strategy was optimized on a validated computer simulation environment for the critically ill [31] before study commencement to ensure favorable outcomes.

Our study is the first randomized controlled trial to evaluate fully automated closed-loop glucose control based on subcutaneous continuous glucose monitoring in critically ill patients. Another closed-loop study used subcutaneous glucose levels but was limited by a lack of a randomized design, a system that was able to control glucose in only one of five studied patients without manual interventions and relatively poor sensor performance, with 64% of values within 20% of reference glucose levels [49]. A third study, by using a closed-loop automated system in 208 Japanese intensive care patients, reported 88% of the time that glucose was in the range of 4.0 to 10.0 m*M *without hypoglycemia [50]. However, this was a retrospective observational study and used the STG-22 system (Nikkiso, Tokyo, Japan), which relies on continuous intravenous glucose measurements drawing 2 ml of blood per hour and is expensive [51], limiting its prolonged and wider use.

We initialized the closed-loop system by using approximate body weight and a reference glucose level. The system did not require information about nutritional intake and was able to respond to rapid changes in caloric and carbohydrate intake, even though a 15-minute lag exists between blood and Navigator sensor glucose levels [52]. When sensor glucose was unavailable during warm-up or for other technical reasons, the system used hourly arterial blood glucose without interruptions to insulin/dextrose delivery.

We increased accuracy of the subcutaneous continuous glucose monitor by calibrating with arterial blood glucose at a frequency higher than recommended by the manufacturer. During the first 24 hours, calibration occurred on average every 2.5 hours, and during the second 24 hours, every 3.5 hours. This is comparable with the present nurse workload. Benefits of subcutaneous glucose monitoring compared with intravenous measurements include reduced invasiveness, obviating the need for dedicated venous placement and a risk of contamination from dextrose or other medications that may interfere with glucose measurements. The risk of infection and thrombosis is lower with the subcutaneous route. The subcutaneous sensor placement was not associated with any complications.

The strengths of our study include the randomized controlled study design, the use of hourly arterial blood glucose to assess outcomes, comparability of the patient groups, and comparable nutrition and treatment modalities. Study limitations include a small sample size, a single-center study design involving a subspecialized patient population, and short study duration, which limits generalizability but does not affect the main study outcomes. The control achieved by using the sliding-scale protocol appears suboptimal and reflects the fear of hypoglycemia in the post-NICE-SUGAR era. Comparisons with other standard insulin-infusion protocols would be beneficial.

In conclusion, automated closed-loop therapy, based on subcutaneous continuous glucose measurements, is a safe and efficacious approach for glucose control in critically ill adults. Larger and longer-duration studies are warranted to assess system performance. Apart from providing a tangible treatment option, closed-loop systems may contribute important insights into the ongoing debate about glucose targets by providing the means to achieve uniform and safe outcomes in comparability studies.

## Key messages

• Fully automated closed-loop glucose control based on subcutaneous sensor glucose is feasible.

• Closed-loop treatment provided safe, effective, and consistent glucose control without increasing the risk of hypoglycemia in a small group of patients over a 48-hour period.

• Closed-loop treatment was superior to a local sliding-scale treatment protocol.

• Nurse intervention is not required during closed-loop treatment, apart from calibrating a subcutaneous glucose monitor.

• Automated administration of dextrose augmented the ability of closed-loop treatment to avoid low glucose levels.

## Abbreviations

CGM: Continuous glucose monitoring; CL: closed-loop; NCCU: Neurosciences Critical Care Unit at Addenbrooke's Hospital: Cambridge: UK.

## Competing interests

LL, SWE, HT, KC, JMA, KK, MEW, MN, JM, and RB have no conflicts of interest. RH reports having received speaker honoraria from Minimed Medtronic, Lifescan, Eli Lilly, and Novo Nordisk, serving on advisory panel for Animas and Minimed Medtronic, receiving license fees from BBraun; and having served as a consultant to BBraun and Profil. MLE reports having received speaker honoraria/travel support from Abbott Diabetes Care, Animas, Medtronic, and Eli Lilly, and serving on advisory boards for Medtronic, Roche, and Cellnovo.

## Authors' contributions

RH conceptualized the study, is the guarantor, and had full access to all the data in the study. RH, LL, RB, SWE, and MLE codesigned the study. LL, HT, SWE, KC, and JMA were responsible for patient screening and enrolment and informed consent. LL, HT, KC, JMA, and KK provided patient care and contributed to acquisition of data. RH designed and implemented the algorithm. RH, MN, MEW, and JM developed and validated the closed-loop system including the conduct of simulation studies. LL and MN carried out the data and statistical analyses. LL and RH drafted the manuscript. All authors critically revised the manuscript and approved the final version of the report.

## References

[B1] Kavanagh BP McCowen KC Clinical practice: glycemic control in the ICU N Engl J Med 2010 17 26 2540 2546 10.1056/NEJMcp1001115 21175316

[B2] Krinsley JS Understanding glycemic control in the critically ill: three domains are better than one Intensive Care Med 2011 17 3 382 384 10.1007/s00134-010-2110-3 21210079

[B3] Krinsley JS Association between hyperglycemia and increased hospital mortality in a heterogeneous population of critically ill patients Mayo Clin Proc 2003 17 12 1471 1478 10.4065/78.12.1471 14661676

[B4] Bochicchio GV Joshi M Bochicchio KM Pyle A Johnson SB Meyer W Lumpkins K Scalea TM Early hyperglycemic control is important in critically injured trauma patients J Trauma 2007 17 6 1353 1358 discussion, 1358-1359 10.1097/TA.0b013e31815b83c4 18212660

[B5] Bagshaw SM Egi M George C Bellomo R Australia New Zealand Intensive Care Society Database Management C Early blood glucose control and mortality in critically ill patients in Australia Crit Care Med 2009 17 2 463 470 10.1097/CCM.0b013e318194b097 19114915

[B6] NICE-SUGAR Study Investigators Finfer S Liu B Chittock DR Norton R Myburgh JA McArthur C Mitchell I Foster D Dhingra V Henderson WR Ronco JJ Bellomo R Cook D McDonald E Dodek P Hebert PC Heyland DK Robinson BG Hypoglycemia and risk of death in critically ill patients N Engl J Med 2012 17 12 1108 1118 22992074 10.1056/NEJMoa1204942

[B7] Hermanides J Bosman RJ Vriesendorp TM Dotsch R Rosendaal FR Zandstra DF Hoekstra JB DeVries JH Hypoglycemia is associated with intensive care unit mortality Crit Care Med 2010 17 6 1430 1434 10.1097/CCM.0b013e3181de562c 20386307

[B8] Egi M Bellomo R Stachowski E French CJ Hart G Variability of blood glucose concentration and short-term mortality in critically ill patients Anesthesiology 2006 17 2 244 252 10.1097/00000542-200608000-00006 16871057

[B9] Krinsley JS Glycemic variability: a strong independent predictor of mortality in critically ill patients Crit Care Med 2008 17 11 3008 3013 10.1097/CCM.0b013e31818b38d2 18824908

[B10] Badawi O Waite MD Fuhrman SA Zuckerman IH Association between intensive care unit-acquired dysglycemia and in-hospital mortality Crit Care Med 2012 17 12 3180 3188 10.1097/CCM.0b013e3182656ae5 22971590

[B11] Dungan KM Braithwaite SS Preiser JC Stress hyperglycaemia Lancet 2009 17 9677 1798 1807 10.1016/S0140-6736(09)60553-5 19465235 PMC3144755

[B12] Van den Berghe G Wouters P Weekers F Verwaest C Bruyninckx F Schetz M Vlasselaers D Ferdinande P Lauwers P Bouillon R Intensive insulin therapy in critically ill patients N Engl J Med 2001 17 19 1359 1367 10.1056/NEJMoa011300 11794168

[B13] Van den Berghe G Wilmer A Hermans G Meersseman W Wouters PJ Milants I Van Wijngaerden E Bobbaers H Bouillon R Intensive insulin therapy in the medical ICU N Engl J Med 2006 17 5 449 461 10.1056/NEJMoa052521 16452557

[B14] NICE-SUGAR Study Investigators Finfer S Chittock DR Su SY Blair D Foster D Dhingra V Bellomo R Cook D Dodek P Henderson WR Hebert PC Heritier S Heyland DK McArthur C McDonald E Mitchell I Myburgh JA Norton R Potter J Robinson BG Ronco JJ Intensive versus conventional glucose control in critically ill patients N Engl J Med 2009 17 13 1283 1297 19318384 10.1056/NEJMoa0810625

[B15] Preiser JC Devos P Ruiz-Santana S Melot C Annane D Groeneveld J Iapichino G Leverve X Nitenberg G Singer P Wernerman J Joannidis M Stecher A Chiolero R A prospective randomised multi-centre controlled trial on tight glucose control by intensive insulin therapy in adult intensive care units: The Glucontrol Study Intensive Care Med 2009 17 10 1738 1748 10.1007/s00134-009-1585-2 19636533

[B16] Griesdale DE de Souza RJ van Dam RM Heyland DK Cook DJ Malhotra A Dhaliwal R Henderson WR Chittock DR Finfer S Talmor D Intensive insulin therapy and mortality among critically ill patients: a meta-analysis including NICE-SUGAR study data CMAJ 2009 17 8 821 827 10.1503/cmaj.090206 19318387 PMC2665940

[B17] Van den Berghe G Intensive insulin therapy in the ICU: reconciling the evidence Nature Rev Endocrinol 2012 17 6 374 378 22310851 10.1038/nrendo.2012.14

[B18] Van den Berghe G Schetz M Vlasselaers D Hermans G Wilmer A Bouillon R Mesotten D Clinical review: intensive insulin therapy in critically ill patients: NICE-SUGAR or Leuven blood glucose target? J Clin Endocrinol Metab 2009 17 9 3163 3170 10.1210/jc.2009-0663 19531590

[B19] Van Herpe T De Moor B Van den Berghe G Towards closed-loop glycaemic control Best Pract Res Clin Anaesthesiol 2009 17 1 69 80 10.1016/j.bpa.2008.07.003 19449617

[B20] Aragon D Evaluation of nursing work effort and perceptions about blood glucose testing in tight glycemic control Am J Crit Care 2006 17 4 370 377 16823014

[B21] Juvenile Diabetes Research Foundation Continuous Glucose Monitoring Study Group Tamborlane WV Beck RW Bode BW Buckingham B Chase HP Clemons R Fiallo-Scharer R Fox LA Gilliam LK Hirsch IB Huang ES Kollman C Kowalski AJ Laffel L Lawrence JM Lee J Mauras N O'Grady M Ruedy KJ Tansey M Tsalikian E Weinzimer S Wilson DM Wolpert H Wysocki T Xing D Continuous glucose monitoring and intensive treatment of type 1 diabetes N Engl J Med 2008 17 14 1464 1476 18779236 10.1056/NEJMoa0805017

[B22] Pickup JC Freeman SC Sutton AJ Glycaemic control in type 1 diabetes during real time continuous glucose monitoring compared with self monitoring of blood glucose: meta-analysis of randomised controlled trials using individual patient data BMJ 2011 17 d3805 10.1136/bmj.d3805 21737469 PMC3131116

[B23] Corstjens AM Ligtenberg JJ van der Horst IC Spanjersberg R Lind JS Tulleken JE Meertens JH Zijlstra JG Accuracy and feasibility of point-of-care and continuous blood glucose analysis in critically ill ICU patients Crit Care 2006 17 5 R135 10.1186/cc5048 16981981 PMC1751062

[B24] Siegelaar SE Barwari T Hermanides J Stooker W van der Voort PH DeVries JH Accuracy and reliability of continuous glucose monitoring in the intensive care unit: a head-to-head comparison of two subcutaneous glucose sensors in cardiac surgery patients Diabetes Care 2011 17 3 e31 10.2337/dc10-1882 21357356 PMC3041230

[B25] Holzinger U Warszawska J Kitzberger R Herkner H Metnitz PG Madl C Impact of shock requiring norepinephrine on the accuracy and reliability of subcutaneous continuous glucose monitoring Intensive Care Med 2009 17 8 1383 1389 10.1007/s00134-009-1471-y 19350213

[B26] Hovorka R Closed-loop insulin delivery: from bench to clinical practice Nature Rev Endocrinol 2011 17 7 385 395 10.1038/nrendo.2011.32 21343892

[B27] Scott NW McPherson GC Ramsay CR Campbell MK The method of minimization for allocation to clinical trials. a review Control Clin Trials 2002 17 6 662 674 10.1016/S0197-2456(02)00242-8 12505244

[B28] Minim: allocation by minimisation in clinical trials http://www-users.york.ac.uk/~mb55/guide/minim.htm

[B29] Geoffrey M Brazg R Richard W FreeStyle Navigator Continuous Glucose Monitoring System with TRUstart algorithm, a 1-hour warm-up time J Diabetes Sci Technol 2011 17 1 99 106 21303631 10.1177/193229681100500114 PMC3045239

[B30] Bequette B A critical assessment of algorithms and challenges in the development of a closed-loop artificial pancreas Diabetes Technol Ther 2005 17 1 28 47 10.1089/dia.2005.7.28 15738702

[B31] Wilinska ME Blaha J Chassin LJ Cordingley JJ Dormand NC Ellmerer M Haluzik M Plank J Vlasselaers D Wouters PJ Hovorka R Evaluating glycemic control algorithms by computer simulations Diabetes Technol Ther 2011 17 7 713 722 10.1089/dia.2011.0016 21488803

[B32] Hovorka R Shojaee-Moradie F Carroll PV Chassin LJ Gowrie IJ Jackson NC Tudor RS Umpleby AM Jones RH Partitioning glucose distribution/transport, disposal, and endogenous production during IVGTT Am J Physiol Endocrinol Metab 2002 17 5 E992 1007 11934663 10.1152/ajpendo.00304.2001

[B33] Qaseem A Humphrey LL Chou R Snow V Shekelle P Clinical Guidelines Committee of the American College of P Use of intensive insulin therapy for the management of glycemic control in hospitalized patients: a clinical practice guideline from the American College of Physicians Ann Intern Med 2011 17 4 260 267 10.7326/0003-4819-154-4-201102150-00007 21320941

[B34] American Diabetes A Standards of medical care in diabetes: 2012 Diabetes Care 2012 17 Suppl 1 S11 63 22187469 10.2337/dc12-s011 PMC3632172

[B35] Jacobi J Bircher N Krinsley J Agus M Braithwaite SS Deutschman C Freire AX Geehan D Kohl B Nasraway SA Rigby M Sands K Schallom L Taylor B Umpierrez G Mazuski J Schunemann H Guidelines for the use of an insulin infusion for the management of hyperglycemia in critically ill patients Crit Care Med 2012 17 12 3251 3276 10.1097/CCM.0b013e3182653269 23164767

[B36] Siegelaar SE Hermanides J Oudemans-van Straaten HM van der Voort PH Bosman RJ Zandstra DF DeVries JH Mean glucose during ICU admission is related to mortality by a U-shaped curve in surgical and medical patients: a retrospective cohort study Critical Care 2010 17 6 R224 10.1186/cc9369 21143980 PMC3219982

[B37] Balkin M Mascioli C Smith V Alnachawati H Mehrishi S Saydain G Slone H Alessandrini J Brown L Achieving durable glucose control in the intensive care unit without hypoglycaemia: a new practical IV insulin protocol Diabetes Metab Res Rev 2007 17 1 49 55 10.1002/dmrr.673 16874843

[B38] Goldberg PA Siegel MD Sherwin RS Halickman JI Lee M Bailey VA Lee SL Dziura JD Inzucchi SE Implementation of a safe and effective insulin infusion protocol in a medical intensive care unit Diabetes Care 2004 17 2 461 467 10.2337/diacare.27.2.461 14747229

[B39] Kanji S Singh A Tierney M Meggison H McIntyre L Hebert PC Standardization of intravenous insulin therapy improves the efficiency and safety of blood glucose control in critically ill adults Intensive Care Med 2004 17 5 804 810 10.1007/s00134-004-2252-2 15127193

[B40] Chase JG Shaw G Le Compte A Lonergan T Willacy M Wong XW Lin J Lotz T Lee D Hann C Implementation and evaluation of the SPRINT protocol for tight glycaemic control in critically ill patients: a clinical practice change Critical Care 2008 17 2 R49 10.1186/cc6868 18412978 PMC2447603

[B41] Davidson PC Steed RD Bode BW Glucommander: a computer-directed intravenous insulin system shown to be safe, simple, and effective in 120,618 h of operation Diabetes Care 2005 17 10 2418 2423 10.2337/diacare.28.10.2418 16186273

[B42] Vogelzang M Zijlstra F Nijsten MW Design and implementation of GRIP: a computerized glucose control system at a surgical intensive care unit BMC Med Informat Decision Making 2005 17 38 10.1186/1472-6947-5-38 PMC1334184 16359559

[B43] Plank J Blaha J Cordingley J Wilinska M Chassin L Morgan C Squire S Haluzik M Kremen J Svacina S Toller W Plasnik A Ellmerer M Hovorka R Pieber T Multicentric, randomized, controlled trial to evaluate blood glucose control by the model predictive control algorithm versus routine glucose management protocols in intensive care unit patients Diabetes Care 2006 17 2 271 276 10.2337/diacare.29.02.06.dc05-1689 16443872

[B44] Pachler C Plank J Weinhandl H Chassin LJ Wilinska ME Kulnik R Kaufmann P Smolle KH Pilger E Pieber TR Ellmerer M Hovorka R Tight glycaemic control by an automated algorithm with time-variant sampling in medical ICU patients Intensive Care Med 2008 17 7 1224 1230 10.1007/s00134-008-1033-8 18297268

[B45] Blaha J Kopecky P Matias M Hovorka R Kunstyr J Kotulak T Lips M Rubes D Stritesky M Lindner J Semrad M Haluzik M Comparison of three protocols for tight glycemic control in cardiac surgery patients Diabetes Care 2009 17 5 757 761 10.2337/dc08-1851 19196894 PMC2671097

[B46] Cordingley J Vlasselaers D Dormand N Wouters P Squire S Chassin L Wilinska M Morgan C Hovorka R Van den Berghe G Intensive insulin therapy: enhanced Model Predictive Control algorithm versus standard care Intensive Care Med 2009 17 1 123 128 10.1007/s00134-008-1236-z 18661120

[B47] Hovorka R Kremen J Blaha J Matias M Anderlova K Bosanska L Roubicek T Wilinska ME Chassin LJ Svacina S Haluzik M Blood glucose control by a model predictive control algorithm with variable sampling rate versus a routine glucose management protocol in cardiac surgery patients: a randomized controlled trial J Clin Endocrinol Metab 2007 17 8 2960 2964 10.1210/jc.2007-0434 17550955

[B48] Van Herpe T Mesotten D Wouters PJ Herbots J Voets E Buyens J De Moor B Van den Berghe G LOGIC-insulin algorithm-guided versus nurse-directed blood glucose control during critical illness: the LOGIC-1 single-center, randomized, controlled clinical trial Diabetes Care 2013 17 2 188 194 10.2337/dc12-0584 22961576 PMC3554274

[B49] Chee F Fernando T van Heerden PV Closed-loop glucose control in critically ill patients using continuous glucose monitoring system (CGMS) in real time IEEE Trans Inf Technol Biomed 2003 17 1 43 53 10.1109/TITB.2003.808509 12670018

[B50] Yatabe T Yamazaki R Kitagawa H Okabayashi T Yamashita K Hanazaki K Yokoyama M The evaluation of the ability of closed-loop glycemic control device to maintain the blood glucose concentration in intensive care unit patients Crit Care Med 2011 17 3 575 578 10.1097/CCM.0b013e318206b9ad 21178768

[B51] Okabayashi T Kozuki A Sumiyoshi T Shima Y Technical challenges and clinical outcomes of using a closed-loop glycemic control system in the hospital J Diabetes Sci Technol 2013 17 1 238 246 23439182 10.1177/193229681300700129 PMC3692238

[B52] Garg SK Voelmle M Gottlieb PA Time lag characterization of two continuous glucose monitoring systems Diabetes Res Clin Pract 2010 17 3 348 353 10.1016/j.diabres.2009.11.014 20022127

